# Neuropsychiatric symptoms and lifelong mental activities in cerebral amyloid angiopathy – a cross-sectional study

**DOI:** 10.1186/s13195-024-01519-3

**Published:** 2024-09-04

**Authors:** Marc Dörner, Anthony Tyndall, Nicolin Hainc, Roland von Känel, Katja Neumann, Sebastian Euler, Frank Schreiber, Philipp Arndt, Erelle Fuchs, Cornelia Garz, Wenzel Glanz, Michaela Butryn, Jan Ben Schulze, Sarah Lavinia Florence Schiebler, Anna-Charlotte John, Annkatrin Hildebrand, Andreas B. Hofmann, Lena Machetanz, Johannes Kirchebner, Pawel Tacik, Alexander Grimm, Robin Jansen, Marc Pawlitzki, Solveig Henneicke, Jose Bernal, Valentina Perosa, Emrah Düzel, Sven G. Meuth, Stefan Vielhaber, Hendrik Mattern, Stefanie Schreiber

**Affiliations:** 1 German Center for Neurodegenerative Diseases (DZNE) within the Helmholtz Association, 39120 Magdeburg, Germany; 2 Department of Consultation-Liaison-Psychiatry and Psychosomatic Medicine, University Hospital Zurich, University of Zurich, Culmannstrasse 8, Zurich, 8091 Switzerland; 3 Department of Neuroradiology, Clinical Neuroscience Center, University Hospital Zurich, University of Zurich, Zurich, 8091 Switzerland; 4 Department of Neurology, Otto-von-Guericke University, 39120 Magdeburg, Germany; 5 Department of Neuroradiology, Otto-von-Guericke University, 39120 Magdeburg, Germany; 6 Institute of Cognitive Neurology and Dementia Research, Otto-von-Guericke University, 39120 Magdeburg, Germany; 7 Department of Psychiatry, Psychotherapy, and Psychosomatics, University Hospital of Psychiatry Zurich, University of Zurich, Zurich, 8032 Switzerland; 8 Department of Forensic Psychiatry, University Hospital of Psychiatry Zurich, University of Zurich, Zurich, 8032 Switzerland; 9 Department of Parkinson’s Disease, Sleep and Movement Disorders, University Hospital Bonn, 53127 Bonn, Germany; 10 German Center for Neurodegenerative Diseases (DZNE) within the Helmholtz Association, 53127 Bonn, Germany; 11 Center for Neurology, Tuebingen University Hospital and Hertie-Institute for Clinical Brain Research, Eberhard Karls University, 72076 Tuebingen, Tuebingen, Germany; 12 Department of Neurology, Heinrich Heine University, 40225 Düsseldorf, Germany; 13 J. Philip Kistler Stroke Research Center, Massachusetts General Hospital, Boston, MA 02114 USA; 14 Center for Behavioural Brain Sciences (CBBS), 39120 Magdeburg, Germany; 15 Biomedical Magnetic Resonance, Otto-von-Guericke University, 39120 Magdeburg, Germany

**Keywords:** Cerebral amyloid angiopathy, Neuropsychiatric symptoms, Depression, Lifelong mental activities, Alzheimer’s disease, White matter hyperintensities, Magnetic resonance imaging

## Abstract

**Background:**

While several studies in cerebral amyloid angiopathy (CAA) focus on cognitive function, data on neuropsychiatric symptoms (NPS) and lifelong mental activities in these patients are scarce. Since NPS are associated with functional impairment, faster cognitive decline and faster progression to death, replication studies in more diverse settings and samples are warranted.

**Methods:**

We prospectively recruited *n* = 69 CAA patients and *n* = 18 cognitively normal controls (NC). The number and severity of NPS were assessed using the Alzheimer’s Disease (AD) Assessment Scale’s (ADAS) noncognitive subscale. We applied different regression models exploring associations between NPS number or severity and group status (CAA vs. NC), CAA severity assessed with magnetic resonance imaging (MRI) or cognitive function (Mini-Mental State Examination (MMSE), ADAS cognitive subscale), adjusting for age, sex, years of education, arterial hypertension, AD pathology, and apolipoprotein E status. Mediation analyses were performed to test indirect effects of lifelong mental activities on CAA severity and NPS.

**Results:**

Patients with CAA had 4.86 times (95% CI 2.20-10.73) more NPS and 3.56 units (95% CI 1.94–5.19) higher expected NPS severity than NC. Higher total CAA severity on MRI predicted 1.14 times (95% CI 1.01.-1.27) more NPS and 0.57 units (95% CI 0.19–0.95) higher expected NPS severity. More severe white matter hyperintensities were associated with 1.21 times more NPS (95% CI 1.05–1.39) and 0.63 units (95% CI 0.19–1.08) more severe NPS. NPS number (MMSE mean difference − 1.15, 95% CI -1.67 to -0.63; ADAS cognitive mean difference 1.91, 95% CI 1.26–2.56) and severity (MMSE − 0.55, 95% CI -0.80 to -0.30; ADAS cognitive mean difference 0.89, 95% CI 0.57–1.21) predicted lower cognitive function. Greater lifelong mental activities partially mediated the relationship between CAA severity and NPS (indirect effect 0.05, 95% CI 0.0007-0.13), and greater lifelong mental activities led to less pronounced CAA severity and thus to less NPS (indirect effect − 0.08, 95% CI -0.22 to -0.002).

**Discussion:**

This study suggests that NPS are common in CAA, and that this relationship may be driven by CAA severity. Furthermore, NPS seem to be tied to lower cognitive function. However, lifelong mental activities might mitigate the impact of NPS in CAA.

**Supplementary Information:**

The online version contains supplementary material available at 10.1186/s13195-024-01519-3.

## Introduction

Cerebral amyloid angiopathy (CAA) belongs to the group of cerebral small vessel diseases (CSVD) and is characterised by β-amyloid (Aβ) deposits in the walls of small-to-medium-sized arteries, arterioles and venous vessels of the cerebral cortex and pia mater [1]. It is the second most common cause of cerebral haemorrhage, and further associated with ischemic strokes, transient focal neurological episodes (TFNE) and cognitive impairment in a substantial number of older patients [1–3]. Furthermore, CAA is found in at least ten to 40% of older patients and occurs in up to 80% of patients with Alzheimer’s disease (AD) dementia [1, 2]. In vivo, CAA is diagnosed through downstream pathologies, which are detectable on cranial magnetic resonance imaging (cMRI) and confined to lobar regions [4]. Past studies indicated that these pathologies, for example white matter hyperintensities (WMH), microinfarcts, or microbleeds, may be related to cognitive decline and dementia in CAA, even after controlling for concomitant AD pathology [1, 5–9]. Indeed, nearly 80% of CAA patients suffer from mild cognitive impairment (MCI) [10].

While several studies in CAA focus on cognitive function, data on neuropsychiatric symptoms (NPS) in these patients are scarce. NPS are noncognitive behavioural and psychiatric symptoms in neurological disorders [11]. Three case reports identified behavioural problems, personality changes and depression in CAA [12–14]. One neuropathological study observed some overlap in the NPS-profiles of CAA and AD [15]. However, CAA showed only a low prevalence of NPS in this investigation. Four other studies that examined NPS in CAA found a high prevalence of NPS in CAA, such as depression, apathy, and agitation. On the other hand, some of these studies suggested an association between NPS and a higher CAA-burden on cMRI or cognitive function, while others did not. [16–19]. Additionally, these studies did not consider concomitant AD pathology or the apolipoprotein E (APOE) status in CAA, which precludes more disease-specific assumptions. Due to these inconsistent and incomplete findings replication studies are warranted.

Better knowledge on the existence and kind of NPS in CAA may be of great clinical and prognostic relevance: NPS are associated with functional impairment, faster cognitive decline and faster progression to death in neurodegenerative diseases [20–22]. Furthermore, NPS may be treatable through their responsiveness against pharmacological and non-pharmacological interventions. NPS might be even preventable and several recent studies in the healthy elderly have emphasized the protective role of lifelong cognitively stimulating activities, high education and a complex and challenging occupation against depression. A cognitively active lifestyle was thereby always beneficial independently of the lifespan - young adulthood, mid- or late-life – when it was implemented [23, 24]. In CAA, there are so far no studies exploring the role of lifelong mental activities for depression and NPS development.

Thus, we aim to compare (1) the CAA-related NPS profile to that of age- and sex-matched cognitively normal controls (NC). In contrast to the already existing studies [15–19], we included distinct instruments measuring the NPS profile together with the assessment of engagement in lifelong mental activities and additional covariates linked to CAA severity, such as the APOE status and concomitant AD pathology. We also explored (2) the associations of NPS with CAA severity on MRI and (3) with cognitive function. Finally, we assessed (4) indirect mediating effects of lifelong mental activities on CAA severity and NPS.

## Methods

### Study sample

In this cross-sectional study, we prospectively included patients with CAA who were diagnosed and treated at the Department of Neurology at the Otto-von-Guericke University of Magdeburg between 2016 and 2022. Patients had to meet the Boston criteria 1.5 for probable CAA, with clinical and neuroimaging evidence of multiple haemorrhages restricted to lobar, cortical, or cortical-subcortical regions, or alternatively a single lobar, cortical, or cortical-subcortical haemorrhage and focal or disseminated cortical superficial siderosis (CSS). Furthermore, there were no signs of other causes of haemorrhage and patients needed to be older than 54 years to be included in the study [4, 25]. Lumbar puncture was conducted through the diagnostic work-up and cerebrospinal fluid (CSF) was used to apply the biomarker-based “ATN” (Aβ, tau, neurodegeneration) classification to assess a concomitant AD pathology according to the National Institute on Aging - Alzheimer’s Association [26]. Aβ_42/40_ ratio was considered for the determination of Aβ positivity (A+), phosphorylated tau (p-tau) for tau positivity (T+), and total tau (t-tau) or neurofilament light chain (NF-L) for neurodegeneration (N+) (Supplement 1). A + T + N + or A + T + N- were considered as AD pathology [26].

All NC were prospectively recruited from an existing pool of cognitively normal healthy elderly from the German Center for Neurodegenerative Diseases (DZNE). NC had to be free of haemorrhagic cMRI markers, i.e., cerebral microbleeds (CMB), CSSand intracerebral haemorrhage (ICH). A score of ≥ 5 in the Geriatric Depression Scale-Short Form (GDS-SF), indicating at least mild depression, was an exclusion criterion for NC [23].

Participants with CAA or NC were excluded if they were < 55 years, could not undergo cMRI, had other central nervous system diseases, current or past alcohol or drug abuse or if they were not fluent in German. All participants provided written informed consent according to the Declaration of Helsinki. The local Ethics Committee of the Otto-von-Guericke University of Magdeburg, Faculty of Medicine, approved the study (28/16).

### cMRI acquisition and CAA markers

A senior neuroradiologist (A.T.) rated CAA-related neuroimaging markers according to the standards for reporting vascular changes on neuroimaging 2 (STRIVE-2) criteria through standardized 3T cMRI (*n* = 61, 70.2%) or 1.5T cMRI (*n* = 26, 29.8%; Supplement 2) [27, 28]. Neuroimaging markers included haemorrhagic (CMB, CSS, ICH) and non-haemorrhagic markers (WMH, enlarged perivascular spaces (PVS), incidental diffusion-weighted imaging (DWI)-positive lesions, cortical cerebral microinfarcts) as well as global cortical atrophy (GCA). Those were assessed according to previously established scales [29–33]. PVS in the centrum semiovale (CSO) were evaluated according to a validated 4-point visual rating scale (0 = no PVS, 1 = ≤ 10 PVS, 2 = 11–20 PVS, 3 = 21–40 PVS, 4 > 40 PVS; [29]). WMH in deep and periventricular (PV) regions were rated using the Fazekas visual rating scale, giving a maximum of three points for each deep or PV WMH. The total score is calculated by adding the deep and PV WMH scores (0–6 points) [31]. CSS was classified according to the total CSS multifocality score. Each hemisphere is scored separately with a score range of 0 to 2 points, adding the right and left hemisphere scores to a total range of 0 to 4 points, defined as not existent (0), focal (1) or multifocal CSS (≥ 2). One point is given for one sulcus or ≤ 3 immediately adjacent sulci with CSS in each hemisphere and two points are given for ≥ 2 non-adjacent sulci or > 3 immediately adjacent sulci with CSS in each hemisphere [32]. GCA was rated using the Pasquier scale (0 = normal volume/no ventricular enlargement, 1 = opening of sulci/mild ventricular enlargement, 2 = volume loss of gyri/moderate ventricular enlargement, 3 = knife blade atrophy/severe ventricular enlargement) [33]. However, until now, there is no established rating scale considering incidental DWI-positive lesions and cortical cerebral microinfarcts [28]. Therefore, we solely counted the number of incidental DWI-positive lesions and cortical cerebral microinfarcts in the whole brain without applying any specific categorization. To evaluate the overall CAA burden, we applied the total CAA severity score (range 0 to 6 points) through the following scale [34]: one point is given for the presence of (a) 2–4 lobar CMB, (b) high degree CSO PVS (> 20 CSO PVS), (c) deep WMH grade ≥ 2 or PV WMH = 3 or (d) focal CSS, respectively. Two points are given for ≥ 5 lobar CMB and multifocal CSS each.

MRI scans of *n* = 10 participants were, at least several weeks after the initial MRI analyses, chosen randomly and scored a second time by the same neuroradiologist (A.T.), and also by another independent senior neuroradiologist (N.H.). Both raters were blinded to all demographics and clinical information. Intra- and interrater reliability were excellent: kappa_intra_ = 0.863; kappa_inter_ = 0.928.

### Measurements

Demographics, clinical diagnoses, vascular risk factors, including type 2 diabetes (i.e., former diagnosis, and/or intake of antidiabetic medication, and/or HbA1c ≥ 6.5% or fasting plasma glucose level ≥ 7.0 mmol/ L [35]), dyslipidemia (i.e., former diagnosis, and/or lipid lowering medication, and/or abnormal blood levels of total cholesterol (> 5.2 mmol/ L), low density lipoprotein cholesterol (> 2.6 mmol/ L), high density lipoprotein cholesterol (< 1.0 mmol/ L), or triglycerides (> 1.7 mmol/ L) [36]), arterial hypertension (i.e., former diagnosis and/or use of antihypertensive medication for blood pressure control [37]), past or current smoking, and the APOE status [38] were prospectively assessed. To evaluate cognitive status in CAA patients and NC participants, we used the Mini-Mental State Examination (MMSE) and the Clinical Dementia Rating (CDR), dividing participants into cognitively normal subjects (MMSE > 26, and CDR = 0), participants with MCI (MMSE 21–26, and CDR 0.5-1), mild dementia (MMSE 11–20, and CDR 0.5-1) and severe dementia (MMSE ≤ 10, and CDR > 1) [39, 40]. Even though the CDR is categorized into a 5-scale global score, it is not sensitive to distinguish patients with MCI from cases with mild dementia. A recent study suggested that a CDR ≥ 0.5 is capable of distinguishing MCI patients from NC, and that it needs a CDR ≥ 2 to distinguish MCI from AD dementia with sufficient sensitivity and specificity. Additionally, this study demonstrated that the majority of MCI patients had a CDR score of 0.5, and that AD patients’ CDR scores ranged mostly between 0.5 and 1 [41]. In this context, our definition that provides rather a range of CDR scores for different cognitive status profiles than one single CDR value (for MCI and mild dementia cases) together with a precise definition of MMSE scores seems reasonable.

We also utilized the Alzheimer’s Disease Assessment Scale’s cognitive (ADAScog) total score as indicator of cognitive function [42].

NPS were measured using the validated 10-item ADAS noncognitive subscale (ADAS-NC) [42]. The ADAS-NC examines aspects of mood, such as tearfulness and depressed mood, and aspects of behaviour disturbances, including lack of cooperation, pacing and increased activity, as well as other NPS, such as delusions, hallucinations, appetite changes, and concentration deficits. Each of these items is rated on a five-point scale with higher scores representing more severe mood disturbance or behavioural abnormalities. In our study, none of the noncognitive items of the ADAS-NC showed a significant correlation with each other or with the severity of cognitive impairment (MMSE, CDR, ADAScog). For statistical analysis, we used a reduced nine-item subset of the ADAS-NC (ADAS-NC9) items, removing the item “tremors” because it is rather a neurological symptom and not associated with NPS. Our approach is similar to another study [43]. In addition, we used the GDS-SF to evaluate depression in our study sample. The optimal cut-offs of the GDS-SF are ≥ 5 (minor depressive disorder) and ≥ 10 points (major depressive disorder) [23, 44].

We further included the Lifetime of Experiences Questionnaire (LEQ), which assesses complex lifelong mental activities. The LEQ evaluates cognitively stimulating activities during three life stages, ranging from young adulthood (13–30 years), to mid-life (31–65 years) and late-life (65 years onwards) [45].

### Statistical procedures

For statistical analysis, we used IBM SSPSS Statistics for Windows, Version 29 (Armonk, NY: IBM Corp). Chi-square and Fisher’s exact test were used to compare non-normally distributed categorical variables. Non-normally distributed continuous and ordinal variables were compared with a Mann-Whitney U or Kruskal-Wallis test. The number of NPS was calculated as the sum of all present symptoms of the ADAS-NC9 (range 0–9). The severity of NPS was calculated as the sum of all severity scores of the ADAS-NC9 (0–45), adding the severity scores for all nine NPS.

In a first step, we used a hierarchical generalized linear Poisson regression and a multiple linear regression model to determine the association between group status (CAA vs. NC) as independent variable, demographics (age, sex, education) as covariates and the number or severity of NPS, as respective dependent variables. Other covariates that are related to CAA severity or NPS according to existing literature, were entered sequentially into the regression models: vascular risk factors as binary variables (see Sect. 2.3) [46–50], concomitant AD pathology as binary variable according to the ATN classification (see Sect. 2.1) [1], and APOE status [38]. To assess potential cognitive repercussions of NPS, we utilized a multiple linear regression with NPS number or severity as independent variables, cognition (MMSE and ADAScog total score) as dependent variable, and the other mentioned covariates. Additional generalized linear Poisson and multiple linear regression models tested the effect of MRI markers of CAA severity, on each, NPS number or severity, including MRI field strength (1.5 Tesla or 3 Tesla) as additional covariate. Furthermore, logistic regression was applied to explore associations of CAA MRI markers with the presence or absence of each of the NPS. Collinearity statistics were applied to identify issues of multicollinearity. These showed no evidence of multicollinearity in all regression models [51]. In a second step, we utilized the PROCESS regression path analysis modeling tool for SPSS to estimate mediation effects of lifelong mental activities between cMRI disease severity and NPS number or severity. All regression and mediation models included both CAA patients and NC participants. In these models, data were missing for the covariates AD pathology (25%) and APOE status (9%). These missing data were handled by multiple imputations: In a first step, the missing values were estimated and replaced several times, which results in different data sets with replaced missing values. In a second step, all regression and mediation analyses were conducted on each of the imputed data sets. Finally, these results were consolidated into one estimate applying standard combining rules [52]. This approach is superior against single-imputation strategies because of avoiding creating false precision by a quantification of the uncertainty through multiple plausible values, and thus estimating what the missing values might be. Significance level was set at *p* < 0.05 (two-sided *p*-value) and adjusted for multiple comparisons by post-hoc chi-square testing.

## Results

### Sample

Table 1 illustrates sociodemographic and clinical characteristics of the study sample. Eighty seven participants were included in the study (69 with CAA, 18 with NC), and 100% of our sample was of Caucasian ethnicity. Clinical presentation of CAA patients was transient ischemic attack (TIA) or stroke (*n* = 34, 49.2%), ICH (*n* = 15, 21.7%), cognitive decline (*n* = 12, 17.3%; diagnosed by pathological MMSE/CDR scores and clinical data) or TFNE (*n* = 8, 11.5%). Of note, more than 12 CAA patients suggested a cognitive decline as measured by MMSE and CDR scores (Supplement 3). Yet, in these cases, other diagnoses, such as ICH or stroke, were the leading clinical diagnoses. CSF data were available in 68.1% (*n* = 47) CAA patients, of whom 23.4% (*n* = 11) demonstrated AD pathology. More than 62% (*n* = 42) of the participants with CAA showed some degree of cognitive impairment. In CAA, four out of twelve (33.3%) participants with a clinical presentation of cognitive decline showed AD pathology, and, vice versa, four out of eleven (36.3%) with AD pathology showed a clinical presentation of cognitive decline (Supplement 3 and 4).

CAA patients indicated significantly higher ADAScog scores (mean 16.52 ± 8.08 vs. 5.11 ± 1.32, *p* < 0.001), lower MMSE total scores (mean 24.64 ± 4.07 vs. 28.61 ± 1.03, *p* < 0.001), and higher CDR scores (median 0.5 vs. 0, *p* < 0.001) compared to NC participants. A Kruskal-Wallis test suggested significant differences between CAA subgroups (cognitively normal, MCI, mild dementia) with regard to cognitive scores, i.e., the ADAScog (mean 11.28 ± 4.33 vs. 16.50 ± 6.28 vs. 27.5 ± 7.25), MMSE (mean 28.2 ± 1.08 vs. 24.63 ± 1.40 vs. 17.25 ± 1.91), and CDR (median 0 vs. 0.5 vs. 1) scores (*p* < 0.001).

**Table 1:** Description of the study sample – sociodemographic and clinical characteristics

	Overall (*n* = 87)	Cerebral amyloid angiopathy (*n* = 69)	NC (*n* = 18)	*p*-value (*p* < 0.05) group analysis	*p*-value subgroup analysis*
Age, y	72.68 (7.36)	72.99 (7.80)	71.50 (5.34)	0.372	
Male, n (%)	46 (52.9)	38 (55.10)	8 (44.40)	0.421	
Years of education	13 (8–23)	12.5 (8–23)	15.5 (12–19)	**0.010**	
Diabetes mellitus, n (%)	23 (26.4)	19 (27.50)	4 (22.20)	0.770	
Arterial hypertension, n (%)	73 (83.9)	63 (91.30)	10 (55.60)	**0.001**	
Dyslipidemia, n (%)	44 (50.6)	37 (53.60)	7 (38.90)	0.265	
Smoking, n (%)	*n* = 84 24 (28.57)	*n* = 66 20 (30.30)	*n* = 18 4 (22.20)	0.501	
*APOE status* APOEε3ε3 APOEε3ε2 or APOEε2ε2 APOEε3ε4 or APOEε4ε4 APOEε2ε4	*n* = 79 37 (46.83) 10 (12.65) 30 (37.97) 3 (3.79)	*n* = 61 27 (44.26) 7 (11.47) 26 (42.62) 1 (1.63)	*n* = 18 10 (55.6) 2 (11.10) 4 (22.20) 2 (11.10)	0.143	*p* < 0.00625 0.398 0.965 0.117 0.064
*Cognitive status* Cognitively normal, n (%) Mild cognitive impairment, n (%) Mild dementia, n (%) Severe dementia, n (%)	*n* = 85 43 (50.58) 30 (35.29) 12 (14.11) 0 (0)	*n* = 67 25 (37.31) 30 (44.77) 12 (17.91) 0 (0)	*n* = 18 18 (100) 0 (0) 0 (0) 0 (0)	**< 0.001**	*p* < 0.0083 **< 0.001** **< 0.001** 0.052

Note: Values are mean (standard deviation) or median (range) unless otherwise noted. Significant *p*-values are marked bold. *p*-values are based on chi-square or Fisher’s exact test (if any cell number was < 5) for categorical variables, and the Mann-Whitney U test for continuous variables. *p-value subgroup analysis is based on post-hoc chi-square testing for multiple comparisons. n: number. y: years. NC: Cognitively normal control. APOE: Apolipoprotein E

### CAA is related to greater neuropsychiatric symptom severity and lower lifelong mental activities

More than 43% (*n* = 29) of CAA patients suffered from some degree of depression. The median number of NPS was 2 (range 0–6) in CAA vs. 0 (range 0–1, *p* < 0.001) in NC, and the median total NPS severity was 3 (range 0–11) vs. 0 (range 0–2, *p* < 0.001). Almost all LEQ scores, including education, were significantly lower in CAA compared to NC, indicating a lower variety and frequency of lifelong mental activities in CAA (Table 2). NPS number and severity, depression and LEQ values did not differ between CAA participants with ICH, cognitive decline or concomitant AD pathology, i.e. there was no effect of CAA subgroup on these measures (Supplement 3–5).

**Table 2:** Description of the study sample – NPS and lifelong mental activities

	Overall (*n* = 87)	Cerebral amyloid angiopathy (*n* = 69)	NC (*n* = 18)	*p*-value (*p* < 0.05) group analysis	*p*-value subgroup analysis*
*ADAS-NC*					
ADAS-NC9 total number	1 (0–6)	2 (0–6)	0 (0–1)	**< 0.001**	
ADAS-NC9 total severity	2 (0–11)	3 (0–11)	0 (0–2)	**< 0.001**	
*GDS-SF* No depression, n (%) Minor depressive disorder, n (%) Major depressive disorder, n (%)	*n* = 85 56 (65.88) 26 (30.58) 3 (3.52)	*n* = 67 38 (56.71) 26 (38.80) 3 (4.47)	*n* = 18 18 (100) 0 (0) 0 (0)	**0.001**	*p* < 0.0083 **< 0.001** **0.002** 0.374
*LEQ*					
LEQ YA education	16.50 (7.96)	15.68 (8.20)	19.64 (6.18)	**0.009**	
LEQ YA activities	19.18 (3.93)	19.03 (3.84)	19.76 (4.34)	0.797	
LEQ YA total	36.49 (10.10)	35.47 (10.42)	40.39 (7.87)	**0.012**	
LEQ ML occupation	61.58 (22.75)	59.51 (22.79)	69.52 (21.34)	0.098	
LEQ ML activities	16.78 (3.63)	16.43 (3.61)	18.11 (3.49)	**0.047**	
LEQ ML total	34.34 (8.68)	32.90 (8.25)	39.84 (8.28)	**0.003**	
LEQ LL specific activities	18.71 (5.00)	17.50 (4.59)	23.35 (3.72)	**< 0.001**	
LEQ LL nonspecific activities	14.58 (3.03)	13.93 (2.80)	17.05 (2.64)	**< 0.001**	
LEQ LL total	22.39 (5.32)	21.35 (5.20)	26.40 (3.71)	**< 0.001**	
LEQ total (cognitive reserve)	93.02 (18.63)	89.47 (18.28)	106.63 (13.08)	**< 0.001**	

Note: Values are mean (standard deviation) or median (range) unless otherwise noted. Significant *p*-values are marked bold. *p*-values are based on chi-square or Fisher’s exact test (if any cell number was < 5) for categorical variables, and the Mann-Whitney U test for continuous variables. *p-value subgroup analysis is based on post-hoc chi-square testing for multiple comparisons. NPS: neuropsychiatric symptoms. ADAS-NC9: Alzheimer’s Disease Assessment Scale subscale noncognitive with nine items. GDS-SF: Geriatric Depression Scale-Short Form. LEQ: Lifetime of Experiences Questionnaire. YA: young adult. ML: mid-life. LL: late-life

Table 3 highlights the prevalence of NPS in patients with CAA compared to NC. The most frequent NPS in CAA were lack of concentration (62.3%), depression (43.4%), appetite changes (33.3%) and lack of cooperation (20.2%). CAA subgroups, i.e. ICH, cognitive decline, and AD pathology, did not affect NPS prevalence. Hence, NPS symptoms were similarly prevalent in each of these diagnoses (Supplement 6).

**Table 3:** Prevalence of neuropsychiatric symptoms (NPS) in CAA and NC.

Symptom type	Overall (*n* = 87)	CAA (*n* = 69)	NC (*n* = 18)	*p*-value
Appetite changes	23 (26.43)	23 (33.33)	0	**0.002**
Delusions	1 (1.14)	1 (1.44)	0	1.000
Depression	30 (34.48)	30 (43.47)	0	**< 0.001**
Hallucinations	3 (3.44)	3 (4.34)	0	1.000
Lack of cooperation	14 (16.09)	14 (20.28)	0	**0.035**
Increased activity	6 (6.89)	6 (8.69)	0	0.336
Pacing	4 (4.59)	4 (5.79)	0	0.575
Tearfulness	9 (10.34)	9 (13.04)	0	0.194
Lack of concentration	45 (51.72)	43 (62.31)	2 (11.11)	**< 0.001**

Note: Values are n (%). *p*-values are based on chi-square or Fisher’s exact test (if any cell number was < 5) according to group. Significant *p*-values are marked bold. CAA: cerebral amyloid angiopathy

In Table 4, associations between the number of NPS or severity of NPS and diagnosis, i.e. CAA vs. NC, are depicted. In the first step, diagnosis and demographics together explained 11% (pseudo R^2^, NPS number) or 24% (adjusted R^2^, NPS severity) of the variance of the regression models. In the final model, after the addition of three covariates, arterial hypertension in the second, AD pathology in the third, and APOE status in the fourth step, the variance explained increased to 14% (pseudo R^2^) or remained at 24% (adjusted R^2^). Other vascular risk factors, i.e. diabetes mellitus, dyslipidemia and smoking, were not considered as their prevalence did not differ between CAA and NC (Table 1).

In summary, even after controlling for several confounders, CAA patients demonstrated a more than 4-fold higher NPS incidence (final model: incidence rate ratio 4.86, 95% confidence interval (CI) 2.20-10.73, *p* < 0.001), and more than 3-fold greater NPS severity (final model: mean difference 3.56, 95% CI 1.94–5.19, *p* < 0.001) compared to NC.

We conducted a second regression analysis exploring associations between CAA subgroups (cognitively normal, MCI, mild dementia) as independent variable, NPS number or severity as dependent variable, and the model was adjusted for age, sex, years of education, arterial hypertension, AD pathology, and APOE status. In these models, CAA subgroups had no impact on NPS number or severity (see Supplement 7).

**Table 4:** Multivariable-adjusted associations of CAA diagnosis with the number and severity of NPS.

	Number of NPS		NPS total severity	
	Incidence rate ratio (95% CI)	*p*-value	Estimate (95% CI)	*p*-value
Step 1				
CAA diagnosis Age Female Years of education	6.07 (3.15 to 11.69) 1.05 (1.01 to 1.09) 1.31 (0.76 to 2.26) 0.98 (0.89 to 1.08)	**< 0.001** **0.009** 0.324 0.738	3.32 (1.86 to 4.79) 0.10 (0.02 to 0.18) 0.56 (-0.64 to 1.78) -0.01 (-0.22 to 0.19)	**< 0.001** **0.015** 0.355 0.913
Step 2				
CAA diagnosis Age Female Years of education Arterial hypertension	6.03 (2.99 to 12.19) 1.05 (1.01 to 1.09) 1.31 (0.76 to 2.26) 0.98 (0.89 to 1.08) 1.01 (0.47 to 2.18)	**< 0.001** **0.009** 0.323 0.743 0.964	3.36 (1.78 to 4.94) 0.10 (0.02 to 0.18) 0.56 (-0.66 to 1.78) -0.01 (-0.22 to 0.19) -0.11 (-1.83 to 1.61)	**< 0.001** **0.016** 0.366 0.902 0.898
Step 3				
CAA diagnosis Age Female Years of education Arterial hypertension AD pathology	4.43 (1.97 to 9.94) 1.05 (1.01 to 1.09) 1.28 (0.74 to 2.20) 0.98 (0.89 to 1.07) 1.09 (0.51 to 2.34) 1.15 (0.50 to 2.64)	**< 0.001** **0.009** 0.366 0.720 0.816 0.726	3.34 (1.75 to 4.92) 0.09 (0.01 to 0.18) 0.57 (-0.65 to 1.80) -0.01 (-0.22 to 0.19) -0.06 (-1.79 to 1.66) 0.74 (-1.13 to 2.62)	**< 0.001** **0.025** 0.355 0.883 0.944 0.433
Step 4				
CAA diagnosis Age Female Years of education Arterial hypertension AD pathology	4.86 (2.20 to 10.73) 1.05 (1.01 to 1.08) 1.26 (0.74 to 2.13) 0.98 (0.89 to 1.07) 1.08 (0.52 to 2.26) 1.50 (0.62 to 3.57)	**< 0.001** **0.009** 0.380 0.746 0.823 0.360	3.56 (1.94 to 5.19) 0.09 (0.00 to 0.18) 0.58 (-0.65 to 1.82) 0.00 (-0.22 to 0.21) -0.08 (-1.81 to 1.65) 1.10 (-0.92 to 3.13)	**< 0.001** **0.031** 0.349 0.972 0.927 0.281
*APOE status*				
Ho/he APOEε4 Ho/he APOEε2 APOEε4 and APOEε2	0.48 (0.26 to 0.90) 0.51 (0.22 to 1.22) 1.52 (0.36 to 6.30)	**0.022** 0.135 0.562	-0.90 (-2.45 to 0.63) -1.01 (-3.17 to 1.13) 0.41 (-3.01 to 3.85)	0.247 0.350 0.809

Note: CI: confidence interval. AD: Alzheimer’s disease. Ho/he: homozygote or heterozygote. Significant *p*-values are marked bold

### CAA severity predicts neuropsychiatric symptom severity

Regression models including MRI markers known to contribute most to CAA severity [34] suggested that more severe WMH and a higher CAA total score predict higher numbers (incidence rate ratio 1.21, 95% CI 1.05–1.39, *p* = 0.006, respectively, incidence rate ratio 1.14, 95% CI 1.01–1.27, *p* = 0.022) and a greater severity of NPS (mean difference 0.63, 95% CI 0.19–1.08, *p* = 0.005, respectively, mean difference 0.57, 95% CI 0.19–0.95, *p* = 0.004; Table 5).

**Table 5:** Multivariable-adjusted associations of imaging markers of CAA severity with the number and severity of NPS.

	Number of NPS		NPS total severity	
	Incidence rate ratio (95% CI)	*p*-value	Estimate (95% CI)	*p*-value
*Marker*				
CSO PVS (category 0–4) WMH sum (category 0–6) Lobar CMB count CSS sum (category 0–4) CAA score (0–6)	1.12 (0.84 to 1.49) 1.21 (1.05 to 1.39) 1.00 (0.99 to 1.00) 1.02 (0.88 to 1.20) 1.14 (1.01 to 1.27)	0.426 **0.006** 0.954 0.719 **0.022**	0.13 (-0.84 to 1.10) 0.63 (0.19 to 1.08) 0.00 (-0.12 to 0.02) 0.28 (-0.35 to 0.92) 0.57 (0.19 to 0.95)	0.791 **0.005** 0.473 0.373 **0.004**

Note: Covariates were age, sex, years of education, arterial hypertension, AD pathology, APOE status, and magnetic resonance imaging field strength (3 Tesla or 1.5 Tesla). Significant *p*-values are marked bold. CSO PVS: centrum semiovale enlarged perivascular spaces. WMH: white matter hyperintensities. CMB: cerebral microbleeds. CSS: cortical superficial siderosis

Exploring associations between further MRI markers and NPS number or severity showed that participants with greater GCA were more likely to have a higher NPS number (incidence rate ratio 1.86, 95% CI 1.26–2.73, *p* = 0.002) and more severe NPS (mean difference 2.01, 95% CI 0.63–3.38, *p* = 0.005). In addition, ICH predicted greater NPS severity (mean difference 0.88, 95% CI 0.04–1.73, *p* = 0.040; Supplement 8).

Logistic regression was used to assess differences between MRI markers and the presence or absence of those four NPS, which differed significantly between CAA and NPS (data shown only for significant results). More severe WMH predicted depression (odds ratio (OR) 2.02, 95% CI 1.21–3.39, *p* = 0.007) and impaired concentration (OR 1.69, 95% CI 1.13–2.53, *p* = 0.010). Additionally, participants with higher CAA scores were more likely to be depressed (OR 1.46, 95% CI 1.02–2.10, *p* = 0.039). Other CAA MRI markers were not associated with the presence or absence of single NPS. However, GCA was associated with the presence of depression (OR 4.52, 95% CI 1.17–17.48, *p* = 0.029) and impaired concentration (OR 3.94, 95% CI 1.12–13.84, *p* = 0.032). MRI field strength difference did not reach statistical significance in all regression models that included MRI markers.

### NPS frequency and severity are associated with lower cognitive function

We found that a higher number of NPS and greater NPS severity indicated lower MMSE (mean difference − 1.15, 95% CI -1.67 to -0.63, *p* < 0.001, respectively, mean difference − 0.55, 95% CI -0.80 to -0.30, *p* < 0.001) and higher ADAScog total scores (mean difference 1.91, 95% CI 1.26–2.56, *p* < 0.001, respectively, mean difference 0.89, 95% CI 0.57–1.21, *p* < 0.001).

### Mediation effects of lifelong mental activities on CAA severity and NPS

**Fig. 1:**
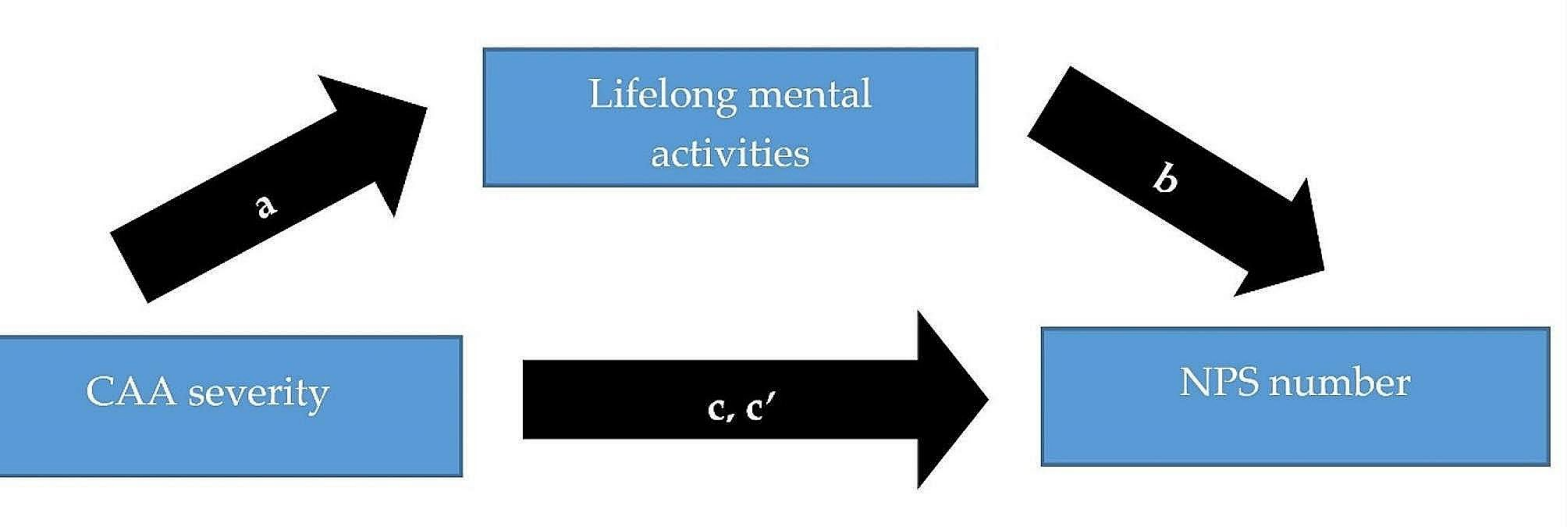
Mediation model testing indirect effects of lifelong mental activities on cerebral amyloid angiopathy (CAA) severity and neuropsychiatric symptom (NPS) number. CAA total score was the independent, NPS number the dependent variable and the lifetime of experiences questionnaire (LEQ) total score the mediator. Covariates were age, sex, years of education, arterial hypertension, Alzheimer’s disease pathology, apolipoprotein E status. **a**: effect of CAA severity on LEQ total score (-1.68, 95% confidence interval (CI) -3.24 to -0.12, p = 0.035). **b**: effect of LEQ total score on NPS number (-0.26, 95% CI -0.05 to -0.002, p = 0.033). **c**’: direct effect of CAA score on NPS number (0.22, 95% CI 0.04–0.39, *p* = 0.014). **c**: total effect of CAA score on NPS number (0.26, 95% CI 0.09–0.44, *p* = 0.003)

Figure 1 illustrates the mediation analysis between CAA severity as independent, NPS number as dependent variable, and the LEQ total score as mediator. The model revealed significant effect sizes of the LEQ total score (i.e. greater lifelong mental activity) as partial mediator mitigating the impact of CAA severity on NPS number (indirect effect 0.05, 95% CI 0.0007-0.13). Using NPS severity as dependent variable instead of NPS number indicated no significant mediating effect of the LEQ total score (indirect effect 0.04, 95% CI -0.002 to 0.12).

We also applied another mediation analysis, reversing the independent variable CAA score and the mediator LEQ total score (Supplement 9). Interestingly, CAA severity significantly partially mediated the relationship between the total LEQ score and NPS number (indirect effect − 0.08, 95% CI -0.22 to -0.002). I. e., greater lifelong mental activities led to lower CAA severity and thus to less NPS. There were also significant effects of CAA severity as full mediator between the LEQ total score and NPS severity (indirect effect − 0.09, 95% CI -0.22 to -0.004).

## Discussion

Patients with CAA demonstrated significantly more frequent and more severe NPS than NC. We found that this relationship is driven by CAA severity, i.e., higher CAA scores and more severe WMH. Higher numbers of NPS and more severe NPS were also tied to lower cognitive performance beyond age, education, arterial hypertension, AD pathology and APOE status. Finally, our findings imply that lifelong mental activities mediate the relationship between CAA severity and NPS, and, vice versa, that greater lifelong mental activities lead to less pronounced CAA severity and thus to less NPS.

NPS are associated with functional impairment [11, 20], poorer quality of life [53], greater caregiver burden [54], higher rates of institutionalization [55], and even faster progression to death [22]. Hence, the identification of NPS in CAA is of great clinical importance. However, research on NPS in CAA is limited so far. The identified prevalence of NPS in our study resembles the already existing studies that observed high frequencies of depression and appetite changes, and rather few patients experiencing hallucinations, delusions or motor disturbances [16, 19]. Our findings are also similar to observations in poststroke patients [56, 57].

In line with other studies, the disruption of white matter networks may underlie NPS as neuropathological mechanism [16, 17]. Previous research also indicated that WMH have been associated with late-life depression [58], AD dementia and cognitive decline [59]. Of course, our finding that higher CAA scores in general are associated with NPS points towards other potential mechanisms that might lead to NPS together with matter disruption, such as local brain damage caused by CMB or CSS. Additional analyses suggested that GCA plays a significant role in the development of NPS, which appears in most neurodegenerative diseases. Our finding that ICH is associated with NPS severity is supported by some studies exploring NPS in ICH survivors, including patients with CSVD pathology [18, 60, 61].

A previous study that investigated NPS in CAA did not find any associations between NPS and cognition, concluding that the mechanisms leading to NPS may differ from those leading to cognitive impairment [16]. On the contrary, our study suggests cognitive repercussions of NPS number and severity, which is in line with a more recent study [19]. However, NPS might not be considered as sole marker of cognitive decline or dementia, given that in our study, CAA subgroups (ICH, cognitive decline, and AD pathology) did not affect NPS prevalence and our models were controlled for age, AD pathology and APOE status. Interestingly, other studies pointed out that NPS manifesting in prodromal stages of dementia are of prognostic utility, leading to an increased risk of progression to dementia and increased neuropathological markers of dementia [11, 21].

Therefore, it is most crucial to identify factors that might prevent or mitigate the development of NPS in CAA. Our novel approach, exploring the role of lifelong mental activities for NPS development in CAA, indicated a lower variety and frequency of lifelong mental activities in CAA compared to NC. Of note, greater lifelong mental activities could be identified as potential variable of resilience, mitigating the impact of CAA severity on NPS development as mediator. Furthermore, greater lifelong mental activities were associated with lower CAA severity as potential variable of resistance, and a consequently reduced number of NPS in CAA patients. These findings align with studies showing that higher LEQ scores are associated with better cognitive abilities and lower depressive symptoms in late-life, a higher quality of life and a better functional status during the onset of dementia [23, 24, 45]. Indeed, preventive interventions aiming at increasing the participation in mental activities over the entire lifespan might prove beneficial in decreasing risk of NPS development in later life with its potential devastating consequences. Moreover, NPS are principally treatable through their responsiveness against pharmacological interventions, such as antidepressant therapy. However, one study found that depressed CAA-related ICH survivors were more likely to report resistance to antidepressant treatment compared to non-CAA-related ICH survivors [609]. Future studies should strive to assess which therapy might prove best in treating NPS in CAA.

Of note, only 23% of CAA patients demonstrated a concomitant AD pathology, which is lower than in a recently published study that examined the CSF profile in sporadic CAA and found that 45% of patients had a CSF profile indicative of AD [62]. Concomitant AD pathology was defined different compared to our study (ATN classification: decreased Aβ_42_ instead of Aβ_42/40_ ratio). Moreover, CAA markers that are indicators of late-stage disease were more frequently found in [62], such as disseminated CSS (45–48% vs. 16%) or severe CSO PVS (> 20; 83–97% vs. 58%; Supplement 10). This may explain the observed differences in concomitant AD pathology to a certain degree. Interestingly, in our study, AD pathology did not differ between CAA subgroups, i.e. those with ICH or cognitive decline (Supplement 3 and 5). Additionally, there were no significant differences of CAA markers on MRI, such as CMB, ICH, or CSS, between patients with and without a concomitant AD pathology (Supplement 10), which is in line with [62].This study has several strengths, such as the inclusion of several disease-specific variables and distinct instruments measuring NPS and lifelong mental activities, thus adding new knowledge on NPS in CAA. However, this study also has some limitations. First, it is cross-sectional, and further longitudinal studies should explore causal relations between NPS and other variables. Second, our study was limited to a single centre, which restricts generalizability. Still, our sample of CAA patients is representative, including several CAA subgroups from early- to late-stage disease [63]. Third, our study sample was relatively small, although it equals sample sizes of previous studies [16–19]. Finally, another limitation is that 25% of CSF and 9% of APOE data were missing. Since a complete case analysis would have led to a significant exclusion of participants, less study power, and results restricted to those without missing data that may not even be representative of the original sample, we decided to apply the well-established method of multiple imputations. Although the results of our analyses would have been more precise if all data had been available, multiple imputations can yield a proxy estimate of the information of the missing values and is regarded as one of the most flexible valid missing data approaches [64].

## Conclusions

This study suggests that NPS are common in CAA. However, NPS can be easily screened with established tools. Many of these NPS are potentially treatable through their responsiveness against pharmacological and non-pharmacological interventions. Although NPS seem to be tied to greater CAA severity and lower cognitive function, lifelong mental activities might mitigate the development and impact of NPS in CAA, and thus improving the quality of life in those patients. Future studies should strive to unravel the neuropathological and neuroimaging correlates of NPS in CAA.

## Electronic supplementary material

Below is the link to the electronic supplementary material.

Supplementary Material 1

## Data Availability

The data presented in this study are available on request from the corresponding author.
