# Supplementary material for: Neuropsychiatric symptoms and lifelong mental activities in cerebral amyloid angiopathy – a cross-sectional study
Source: Alzheimers Res Ther. 2024 Sep 4;16:196. doi: 10.1186/s13195-024-01519-3 (PMC11375846; doi:10.1186/s13195-024-01519-3)
Supplement: Supplementary file 1 — Supplementary Material 1 [file 13195_2024_1519_MOESM1_ESM.docx]

**Supplement 1. CSF examination**

No longer than 20 minutes after lumbar puncture, CSF samples were centrifuged at 4 °C, aliquoted and stored at -80 °C until analysis. Thresholds for CSF markers of neurodegeneration were set for Aβ_42/40_ ratio at ≤ 0.05 (until December 2019) or ≤ 0.069 (since January 2020), for t-tau at >350 pg/ml (until December 2019) or >404 pg/ml (since Januray 2020), for neurofilament light chain (NF-L) at >3643 pg/ml, and for p-tau at >70 pg/ml (until December 2019) or >56.5 pg/ml (since January 2020), according to inhouse standards.

**Supplement 2. MRI protocols**

Magnetic resonance imaging (MRI) protocols on Siemens scanners (whole head) included a T_2_-weighted fluid-attenuated inversion recovery (FLAIR) sequence (voxel size 1 mm^3^ isotropic, echo time (TE) 77-395 ms, repetition time (TR) 5000-11000 ms) to evaluate white matter hyperintensities. A T_2_-weighted sequence permitted us to individuate enlarged perivascular spaces and cortical cerebral microinfarcts and to identify global cortical atrophy (voxel size 0.5 × 0.5 × 2 mm^3^ isotropic, TE 74-128 ms, TR 1040-11121 ms). Cerebral microbleeds, cortical superficial siderosis and intracerebral haemorrhage were rated using a susceptibility-weighted 3D gradient-echo pulse sequence (voxel size 1 × 1 × 2 mm^3^ isotropic. TE 14-28 ms, TR 20-1421 ms). Furthermore, a diffusion-weighted imaging sequence was applied to detect incidental DWI-positive lesions (voxel size 1.8 × 1.8 × 5.0 mm^3^, TE 72 ms, TR 8800 ms). Scanning time totalled ∼45 min.

**Supplement 3.** Description of the study sample – CAA with a clinical presentation of cognitive decline vs without clinical presentation of cognitive decline.

|  | **Overall (*n*=69)** | **CAA with a clinical presentation of cognitive decline (*n*=12)** | **CAA without clinical presentation of cognitive decline (*n*=57)** | ***p*-value**  **group analysis (*p*<0.05)** | ***p*-value subgroup analysis*** |
| --- | --- | --- | --- | --- | --- |
| Age, y | 72.99 (7.8) | 72.50 (6.69) | 73.09 (8.06) | 0.794 |  |
| Male, *n* (%) | 38 (55.1) | 7 (58.3) | 31 (54.4) | 0.803 |  |
| Years of education | 12.5 (8-23) | 14.0 (11-20) | 12.0 (8-23) | 0.235 |  |
| Diabetes mellitus, *n* (%) | 19 (27.5) | 2 (16.7) | 17 (29.8) | 0.489 |  |
| Arterial hypertension, *n* (%) | 63 (91.3) | 9 (75.0) | 54 (94.7) | 0.061 |  |
| Dyslipidemia, *n* (%) | 37 (53.6) | 4 (33.3) | 33 (57.9) | 0.121 |  |
| Smoking, *n* (%) | *n*=66  20 (30.30) | *n*=12  3 (25.0) | *n*=54  17 (31.48) | 0.743 |  |
| APOE status  APOEε3ε3  APOEε3ε2 or APOEε2ε2  APOEε3ε4 or APOEε4ε4  APOEε2ε4 | *n*=61  27 (44.26)  7 (11.47)  26 (42.62)  1 (1.63) | *n*=11  4 (36.36)  0 (0)  7 (63.63)  0 (0) | *n*=50  23 (46.0)  7 (14.0)  19 (38.0)  1 (2.0) | 0.385 | *p*<0.00625  0.560  0.187  0.119  0.636 |
| Cognitive status  Cognitively normal, *n* (%)  Mild cognitive impairment, *n* (%)  Mild dementia, *n* (%)  Severe dementia, *n* (%) | *n*=67  25 (37.31)  30 (44.77)  12 (17.91)  0 (0) | *n*=12  0 (0)  9 (75.0)  3 (25.0)  0 (0) | *n*=55  25 (45.45)  21 (38.18)  9 (16.36)  0 (0) | **0.004** | *p*<0.0083  **0.003**  0.020  0.479 |
| AD pathology (yes), *n* (%) | *n*=47  11 (23.40) | *n*=11  4 (36.36) | *n*=36  7 (19.44) | 0.256 |  |
| ADAS-NC  ADASS-NC9 total number | 1 (0-6) | 2 (0-4) | 2 (0-6) | 0.522 |  |
| ADAS-NC9 total severity | 2 (0-11) | 3.5 (0-9) | 2 (0-11) | 0.363 |  |
| GDS-SF  No, *n* (%)  Minor depressive disorder, *n* (%)  Major depressive disorder, *n* (%) | *n*=67  38 (56.71)  26 (38.80)  3 (4.47) | *n*=11  5 (45.45)  5 (45.45)  1 (9.09) | *n*=56  33 (58.92)  21 (37.5)  2 (3.57) | 0.427 | *p*<0.0083  0.409  0.620  0.418 |
| LEQ  LEQ YA education | 15.68 (8.39) | 18.60 (8.74) | 15.16 (8.30) | 0.154 |  |
| LEQ YA activities | 19.03 (3.93) | 17.70 (5.75) | 19.26 (3.53) | 0.131 |  |
| LEQ YA total | 35.47 (10.66) | 37.23 (13.25) | 35.16 (10.24) | 0.886 |  |
| LEQ ML occupation | 59.51 (23.32) | 62.40 (26.56) | 59.00 (22.92) | 0.655 |  |
| LEQ ML activities | 16.43 (3.69) | 16.70 (3.86) | 16.39 (3.69) | 0.871 |  |
| LEQ ML total | 32.90 (8.44) | 32.30 (8.97) | 33.01 (8.42) | 0.775 |  |
| LEQ LL specific activities | 17.50 (4.80) | 16.00 (3.09) | 17.79 (5.03) | 0.183 |  |
| LEQ LL nonspecific activities | 13.93 (2.93) | 13.10 (2.76) | 14.09 (2.96) | 0.460 |  |
| LEQ LL total | 21.35 (5.44) | 19.50 (3.50) | 21.70 (5.70) | 0.229 |  |
| LEQ total (cognitive reserve) | 89.47 (19.14) | 89.03 (21.05) | 89.55 (18.98) | 0.686 |  |

Note: *n*: number. y: years. Values are mean (standard deviation) or median (range) unless otherwise noted. Significant *p*-values are marked bold. *p*-values are based on chi-square or Fisher’s exact test (if any cell number was <5) for categorical variables, and the Mann-Whitney U test for continuous variables. *p-value subgroup analysis is based on post-hoc chi-square testing for multiple comparisons. APOE: Apolipoprotein E. ADAS-NC9: Alzheimer’s Disease Assessment Scale noncognitive subscale with nine items. GDS-SF: Geriatric Depression Scale-Short Form. CAA: cerebral amyloid angiopathy. AD: Alzheimer’s disease. LEQ: Lifetime of Experiences Questionnaire. YA: young adult. ML: mid-life. LL: late-life.

**Supplement 4.** Description of the study sample – CAA with AD pathology vs without AD pathology.

|  | **Overall (*n*=47)** | **CAA with AD pathology (*n*=11)** | **CAA without AD pathology (*n*=36)** | ***p*-value**  **group analysis (*p*<0.05)** | ***p*-value subgroup analysis*** |
| --- | --- | --- | --- | --- | --- |
| Age, y | 72.60 (8.04) | 75.64 (7.99) | 71.67 (7.93) | 0.236 |  |
| Male, *n* (%) | 24 (51.1) | 6 (54.5) | 18 (50.0) | 0.792 |  |
| Years of education | 12.25 (8-21) | 11.0 (10-20) | 125 (8-21) | 0.510 |  |
| Diabetes mellitus, *n* (%) | 9 (19.1) | 3 (27.3) | 6 (16.7) | 0.419 |  |
| Arterial hypertension, *n* (%) | 42 (89.4) | 9 (81.8) | 33 (91.7) | 0.578 |  |
| Dyslipidemia, *n* (%) | 25 (53.2) | 4 (36.4) | 21 (58.3) | 0.201 |  |
| Smoking, *n* (%) | *n*=44  11 (25.0) | *n*=10  2 (20.0) | *n*=34  9 (26.47) | 1.000 |  |
| APOE status  APOEε3ε3  APOEε3ε2 or APOEε2ε2  APOEε3ε4 or APOEε4ε4  APOEε2ε4 | *n*=42  15 (35.71)  6 (14.28)  21 (50.0)  0 (0) | *n*=11  1 (9.1)  0 (0)  10 (90.9)  0 (0) | *n*=31  14 (45.16)  6 (19.35)  11 (35.48)  0 (0) | **0.009** | *p*<0.0083  0.032  0.115  **0.001**  0.636 |
| Cognitive status  Cognitively normal, *n* (%)  Mild cognitive impairment, *n* (%)  Mild dementia, *n* (%)  Severe dementia, *n* (%) | *n*=46  16 (34.78)  20 (43.47)  10 (21.73)  0 (0) | *n*=11  4 (36.4)  5 (45.5)  2 (18.2)  0 (0) | *n*=35  12 (34.28)  15 (42.85)  8 (22.85)  0 (0) | 1.000 | *p*<0.0083  0.899  0.879  0.743 |
| ADAS-NC  ADASS-NC9 total number | 2 (0-6) | 3 (1-4) | 2 (0-6) | 0.416 |  |
| ADAS-NC9 total severity | 3 (0-11) | 5 (1-9) | 2 (0-11) | 0.254 |  |
| GDS-SF  No, *n* (%)  Minor depressive disorder, *n* (%)  Major depressive disorder, *n* (%) | *n*=46  24 (52.17)  20 (43.47)  2 (4.34) | *n*=11  3 (27.3)  7 (63.6)  1 (9.1) | *n*=35  21 (60.0)  13 (37.14)  1 (2.85) | 0.152 | *p*<0.0083  0.058  0.122  0.376 |
| LEQ  LEQ YA education | 15.24 (7.72) | 17.00 (9.80) | 14.74 (7.11) | 0.989 |  |
| LEQ YA activities | 19.15 (4.46) | 19.70 (4.96) | 19.00 (4.37) | 0.799 |  |
| LEQ YA total | 35.19 (10.27) | 37.55 (13.91) | 34.52 (9.12) | 0.840 |  |
| LEQ ML occupation | 60.75 (24.16) | 69.40 (22.95) | 58.28 (24.24) | 0.162 |  |
| LEQ ML activities | 16.93 (3.89) | 16.60 (4.08) | 17.02 (3.89) | 0.717 |  |
| LEQ ML total | 33.33 (8.60) | 34.58 (8.17) | 32.98 (8.80) | 0.600 |  |
| LEQ LL specific activities | 17.57 (4.87) | 18.30 (5.98) | 17.34 (4.56) | 0.551 |  |
| LEQ LL nonspecific activities | 14.00 (2.93) | 14.50 (3.06) | 13.84 (2.93) | 1.000 |  |
| LEQ LL total | 21.64 (5.96) | 23.82 (9.28) | 20.96 (4.47) | 0.611 |  |
| LEQ total (cognitive reserve) | 89.79 (19.61) | 95.92 (24.34) | 87.88 (17.91) | 0.390 |  |

Note: *n*: number. y: years. Values are mean (standard deviation) or median (range) unless otherwise noted. Significant *p*-values are marked bold. *p*-values are based on chi-square or Fisher’s exact test (if any cell number was <5) for categorical variables, and the Mann-Whitney U test for continuous variables. *p-value subgroup analysis is based on post-hoc chi-square testing for multiple comparisons. APOE: Apolipoprotein E. ADAS-NC9: Alzheimer’s Disease Assessment Scale noncognitive subscale with nine items. GDS-SF: Geriatric Depression Scale-Short Form. CAA: cerebral amyloid angiopathy. AD: Alzheimer’s disease. LEQ: Lifetime of Experiences Questionnaire. YA: young adult. ML: mid-life. LL: late-life.

**Supplement 5.** Description of the study sample – CAA with a clinical presentation of ICH vs without ICH.

|  | **Overall (*n*=69)** | **CAA with ICH *(n*=15)** | **CAA without ICH (*n*=54)** | ***p*-value**  **group analysis (*p*<0.05)** | ***p*-value subgroup analysis*** |
| --- | --- | --- | --- | --- | --- |
| Age, y | 72.99 (7.8) | 73.87 (10.22) | 72.74 (7.08) | 0.503 |  |
| Male, *n* (%) | 38 (55.1) | 9 (60.0) | 29 (53.7) | 0.665 |  |
| Years of education | 12.5 (8-23) | 12.0 (8-20) | 12.5 (8-23) | 0.764 |  |
| Diabetes mellitus, *n* (%) | 19 (27.5) | 4 (26.7) | 15 (27.8) | 1.000 |  |
| Arterial hypertension, *n* (%) | 63 (91.3) | 13 (86.7) | 50 (92.6) | 0.604 |  |
| Dyslipidemia, *n* (%) | 37 (53.6) | 7 (46.7) | 30 (55.6) | 0.541 |  |
| Smoking, *n* (%) | *n*=66  20 (30.30) | *n*=15  5 (33.33) | *n*=51  15 (29.41) | 0.759 |  |
| APOE status  APOEε3ε3  APOEε3ε2 or APOEε2ε2  APOEε3ε4 or APOEε4ε4  APOEε2ε4 | *n*=61  27 (44.26)  7 (11.47)  26 (42.62)  1 (1.63) | *n*=13  5 (38.46)  3 (23.07)  5 (38.46)  0 (0) | *n*=48  22 (45.83)  4 (8.33)  21 (43.75)  1 (2.08) | 0.482 | *p*<0.00625  0.635  0.139  0.732  0.599 |
| Cognitive status  Cognitively normal, *n* (%)  Mild cognitive impairment, *n* (%)  Mild dementia, *n* (%)  Severe dementia, *n* (%) | *n*=67  25 (37.31)  30 (44.77)  12 (17.91)  0 (0) | *n*=14  6 (42.85)  5 (35.71)  3 (21.42)  0 (0) | *n*=53  19 (35.84)  25 (47.16)  9 (16.98)  0 (0) | 0.727 | *p*<0.0083  0.629  0.443  0.699 |
| AD pathology (yes), n (%) | *n*=47  11 (23.40) | *n*=9  2 (22.22) | *n*=38  9 (23.68) | 1.000 |  |
| ADAS-NC  ADASS-NC9 total number | 1 (0-6) | 1 (0-4) | 2 (0-6) | 0.236 |  |
| ADAS-NC9 total severity | 2 (0-11) | 2 (0-10) | 3 (0-11) | 0.422 |  |
| GDS-SF  No, *n* (%)  Minor depressive disorder, *n* (%)  Major depressive disorder, *n* (%) | *n*=67  38 (56.71)  26 (38.80)  3 (4.47) | *n*=15  8 (53.3)  6 (40.0)  1 (6.7) | *n*=52  30 (55.55)  20 (37.03)  2 (3.84) | 0.891 | *p*<0.0083  0.764  0.914  0.641 |
| LEQ  LEQ YA education | 15.68 (8.39) | 13.21 (6.86) | 16.34 (8.70) | 0.178 |  |
| LEQ YA activities | 19.03 (3.93) | 18.85 (2.79) | 19.07 (4.20) | 0.819 |  |
| LEQ YA total | 35.47 (10.66) | 32.63 (9.36) | 36.24 (10.93) | 0.304 |  |
| LEQ ML occupation | 59.51 (23.32) | 58.92 (26.98) | 59.67 (22.52) | 0.944 |  |
| LEQ ML activities | 16.43 (3.69) | 16.64 (4.18) | 16.38 (3.59) | 0.881 |  |
| LEQ ML total | 32.90 (8.44) | 33.92 (10.74) | 32.63 (7.81) | 0.655 |  |
| LEQ LL specific activities | 17.50 (4.80) | 17.69 (6.10) | 17.46 (4.48) | 0.980 |  |
| LEQ LL nonspecific activities | 13.93 (2.93) | 13.38 (3.45) | 14.08 (2.80) | 0.325 |  |
| LEQ LL total | 21.35 (5.44) | 22.00 (9.16) | 21.18 (4.10) | 0.715 |  |
| LEQ total (cognitive reserve) | 89.47 (19.14) | 87.84 (25.31) | 89.89 (17.49) | 0.768 |  |

Note: *n*: number. y: years. Values are mean (standard deviation) or median (range) unless otherwise noted. Significant *p*-values are marked bold. *p*-values are based on chi-square or Fisher’s exact test (if any cell number was <5) for categorical variables, and the Mann-Whitney U test for continuous variables. *p-value subgroup analysis is based on post-hoc chi-square testing for multiple comparisons. APOE: Apolipoprotein E. ADAS-NC9: Alzheimer’s Disease Assessment Scale noncognitive subscale with nine items. GDS-SF: Geriatric Depression Scale-Short Form. CAA: cerebral amyloid angiopathy. ICH: intracerebral haemorrhage. AD: Alzheimer’s disease. LEQ: Lifetime of Experiences Questionnaire. YA: young adult. ML: mid-life. LL: late-life.

**Supplement 6.** Prevalence of NPS in CAA subgroups.

| **Symptom type** | **Overall (*n*=69)** | **CAA with ICH (*n*=13)** | **CAA with cognitive decline (*n*=8)** | **CAA with AD pathology (*n*=5)** | **CAA with AD pathology and ICH or cognitive decline overlap (*n*=6)** | **CAA remaining subgroup (*n*=37)** | **p-value (*p*<0.05)^a^** |
| --- | --- | --- | --- | --- | --- | --- | --- |
| Appetite changes | 23 (33.3) | 3 (23.0) | 2 (25.0) | 3 (60) | 1 (16.7) | 14 (37.8) | 0.578 |
| Delusions | 1 (1.4) | 1 (7.6) | 0 (0) | 0 (0) | 0 (0) | 0 (0) | 0.456 |
| Depression | 30 (43.4) | 5 (38.4) | 5 (62.5) | 3 (60.0) | 4 (66.7) | 13 (35.1) | 0.399 |
| Hallucinations | 3 (4.3) | 0 (0) | 0 (0) | 0 (0) | 0 (0) | 3 (8.1) | 0.841 |
| Lack of cooperation | 14 (20.2) | 2 (15.3) | 1 (12.5) | 2 (40.0) | 2 (33.3) | 7 (18.9) | 0.650 |
| Increased activity | 6 (8.6) | 0 (0) | 2 (25.0) | 1 (20.0) | 0 (0) | 3 (8.1) | 0.210 |
| Pacing | 4 (5.7) | 0 (0) | 0 (0) | 0 (0) | 0 (0) | 4 (10.8) | 0.886 |
| Tearfulness | 9 (13.0) | 2 (15.3) | 1 (12.5) | 1 (20.0) | 0 (0) | 5 (13.5) | 0.894 |
| Lack of concentration | 43 (62.3) | 6 (46.1) | 6 (75.0) | 4 (80.0) | 5 (83.3) | 22 (59.5) | 0.593 |

Note: Values are *n* (%). *p*-values are based on chi-square or Fisher’s exact test (if any cell number was <5). Significant *p*-values are marked bold. CAA: cerebral amyloid angiopathy. ICH: intracerebral haemorrhage. AD: Alzheimer’s disease. ^a^ Post-hoc Chi-square testing did not indicate any significant differences between groups.

**Supplement 7.** Multivariable-Adjusted associations of CAA subgroups with the number and severity of NPS.

|  | **Number of NPS** | | **NPS total severity** | |
| --- | --- | --- | --- | --- |
|  | **Incidence rate ratio (95% CI)** | **p-value** | **Estimate (95% CI)** | **p-value** |
| CAA subgroups (cognitively normal/ MCI/ mild dementia)  Age  Female  Years of education  Arterial hypertension  AD pathology  APOE status | 1.32 (0.98 to 1.78)  1.03 (1.00 to 1.07)  2.05 (1.21 to 3.46)  1.04 (0.96 to 1.13)  0.72 (0.33 to 1.55)  1.55 (0.86 to 2.78)  0.77 (0.65 to 0.93) | 0.061  **0.020**  **0.004**  0.312  0.406  0.183  **0.006** | 1.22 (-0.21 to 2.47)  0.15 (0.02 to 0.27)  2.19 (0.16 to 4.22)  0.16 (-0.16 to 0.48)  -0.71 (-3.96 to 2.53)  2.22 (-0.18 to 4.62)  -1.04 (-1.76 to -0.32) | 0.054  **0.018**  **0.035**  0.311  0.657  0.069  **0.006** |

Note: CAA: cerebral amyloid angiopathy. NPS: neuropsychiatric symptoms. MCI: mild cognitive impairment. CI: confidence interval. AD: Alzheimer’s disease. APOE: apolipoprotein E. Significant p-values are marked bold.

**Supplement 8.** Multivariable-Adjusted associations of further imaging markers with the number and severity of NPS.

|  | **Number of NPS** | | **NPS total severity** | |
| --- | --- | --- | --- | --- |
|  | **Incidence rate ratio (95% CI)** | ***p*-value** | **Estimate (95% CI)** | ***p*-value** |
| Marker  Global cortical atrophy (category 0-3)  Cortical cerebral microinfarcts count  Incidental DWI-positive lesions count  ICH count | 1.86 (1.26 to 2.73)  1.32 (0.95 to 1.85)  0.64 (0.40 to 1.01)  1.02 (0.80 to 1.29) | **0.002**  0.094  0.055  0.864 | 2.01 (0.63 to 3.38)  0.52 (-0.73 to 1.79)  -0.94 (-2.52 to 0.63)  0.88 (0.04 to 1.73) | **0.005**  0.408  0.238  **0.040** |

Note: CI: confidence interval. NPS: neuropsychiatric symptoms. DWI: diffusion-weighted imaging. ICH: intracerebral haemorrhage. Covariates were age, sex, years of education, arterial hypertension, Alzheimer’s disease pathology, apolipoprotein E status, and magnetic resonance imaging field strength (3 Tesla or 1.5 Tesla). Significant *p*-values are marked bold.


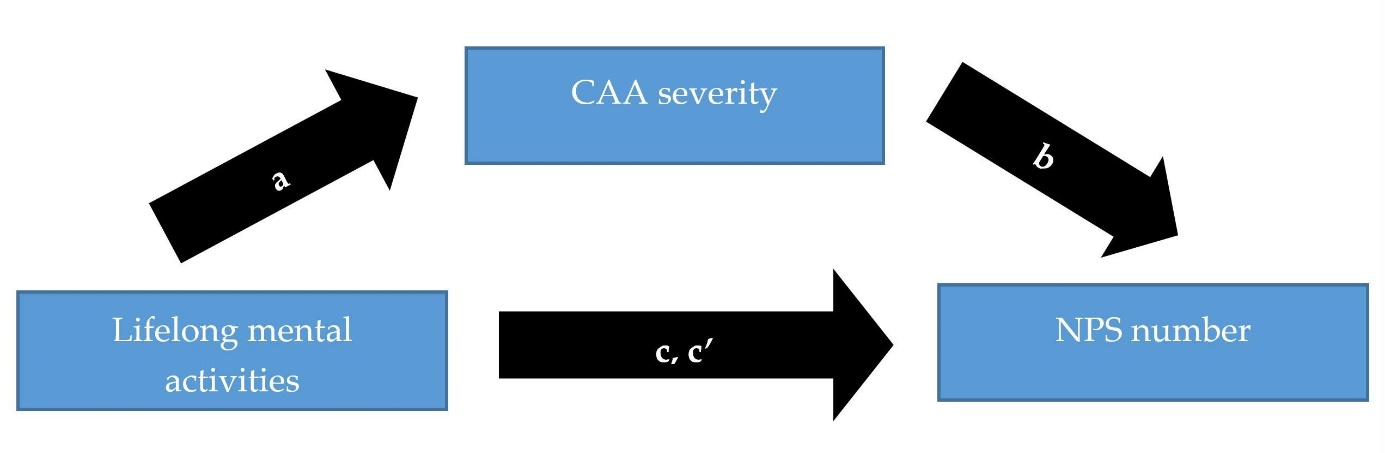


**Supplement 9. Mediation model testing indirect effects of cerebral amyloid angiopathy (CAA) severity on lifelong mental activities and neuropsychiatric symptom (NPS) number.** The lifetime of experiences questionnaire (LEQ) total score was the independent, NPS number the dependent variable and CAA total score the mediator. Covariates were age, sex, years of education, arterial hypertension, Alzheimer’s disease pathology, apolipoprotein E status. a: effect of LEQ total score on CAA severity (-0.03, 95% confidence interval (CI) -0.06 to -0.002, p=0.035). b: effect of CAA severity on NPS number (0.22, 95% CI 0.04-0.39, p=0.014). c’: direct effect of LEQ total score on NPS number (-0.02, 95% CI -0.05 to -0.002, p=0.033). c: total effect of LEQ total score on NPS number (-0.03, 95% CI -0.05 to -0.009, p=0.007).

**Supplement 10. Additional information regarding cerebral amyloid angiopathy (CAA) severity on magnetic resonance imaging (MRI)**

On cranial MRI, 57.9% of all 69 CAA patients demonstrated severe centrum semiovale (CSO) enlarged perivascular spaces (PVS, > 20). In 30.9% of all cases, intracerebral haemorrhage (ICH) could be identified, 15.9% demonstrated focal cortical superficial siderosis (CSS), and 15.8% indicated disseminated CSS. Comparing CAA markers between patients with vs without a concomitant AD pathology, a Mann-Whitney U test did not indicate any significant differences (including CSO PVS, white matter hyperintensities, lobar cerebral microbleeds, CSS, ICH, and the total CAA severity score).
